# Deciphering and targeting host factors to counteract SARS-CoV-2 and coronavirus infections: insights from CRISPR approaches

**DOI:** 10.3389/fgeed.2023.1231656

**Published:** 2023-07-13

**Authors:** Zhifen Cui, Hongyan Wang, Yizhou Dong, Shan-Lu Liu, Qianben Wang

**Affiliations:** ^1^ Department of Pathology, Duke University School of Medicine, Durham, NC, United States; ^2^ Department of Oncological Sciences, Icahn Genomics Institute, Precision Immunology Institute, Tisch Cancer Institute, Friedman Brain Institute, Icahn School of Medicine at Mount Sinai, New York, NY, United States; ^3^ Center for Retrovirus Research, Viruses and Emerging Pathogens Program, Department of Veterinary Biosciences, Infectious Diseases Institute, The Ohio State University, Columbus, OH, United States

**Keywords:** SARS-CoV-2, host factors, CRISPR/Cas9-based screening, CRISPR/Cas13-based gene therapy, coronavirus infections

## Abstract

Severe respiratory syndrome coronavirus 2 (SARS-CoV-2) and other coronaviruses depend on host factors for the process of viral infection and replication. A better understanding of the dynamic interplay between viral pathogens and host cells, as well as identifying of virus-host dependencies, offers valuable insights into disease mechanisms and informs the development of effective therapeutic strategies against viral infections. This review delves into the key host factors that facilitate or hinder SARS-CoV-2 infection and replication, as identified by CRISPR/Cas9-based screening platforms. Furthermore, we explore CRISPR/Cas13-based gene therapy strategies aimed at targeting these host factors to inhibit viral infection, with the ultimate goal of eradicating SARS-CoV-2 and preventing and treating related coronaviruses for future outbreaks.

## Introduction

The COVID-19 pandemic represents a global health crisis that has impacted billions of lives worldwide, posing unprecedented challenges to healthcare systems, economies, and social structures across the globe. Despite the World Health Organization (WHO) declaring the end of COVID-19’s emergence phase on 5 May 2023, the disease continues to pose a global health threat, and the risk of new variants to emerge remains. It is therefore crucial to comprehend the mechanism through which SARS-CoV-2, the causative agent, takes control of the host cell machinery during infection, which shall aid in the development of novel therapeutic approaches. SARS-CoV-2, a member of the Coronaviridae, is an enveloped, positive-sense single-stranded RNA virus with a genome length of approximately 30 kilobases (Fehr and Perlman, 2015; Wang et al., 2020). The infection begins with binding of the virus to receptors and fusing with the membrane, both on the plasma membrane and within endosomes, depending on mutations on the spike protein. This process releases the viral nucleocapsid into cytoplasm, where it undergoes translation to produce viral proteins using genomic RNA as template, then viral replication transcription complexes of virus form on double-membrane vesicles (DMVs), resulting in copies of new viral genome. These copies are then packaged via budding process into mature virions and then released from infected cells (Fung and Liu, 2019; Daniloski et al., 2021). The identification of host factors critical for infection is important for elucidating virus-host interaction and pathogenesis mechanisms, and can offer new strategies for prevention and antiviral therapy.

**FIGURE 1:**
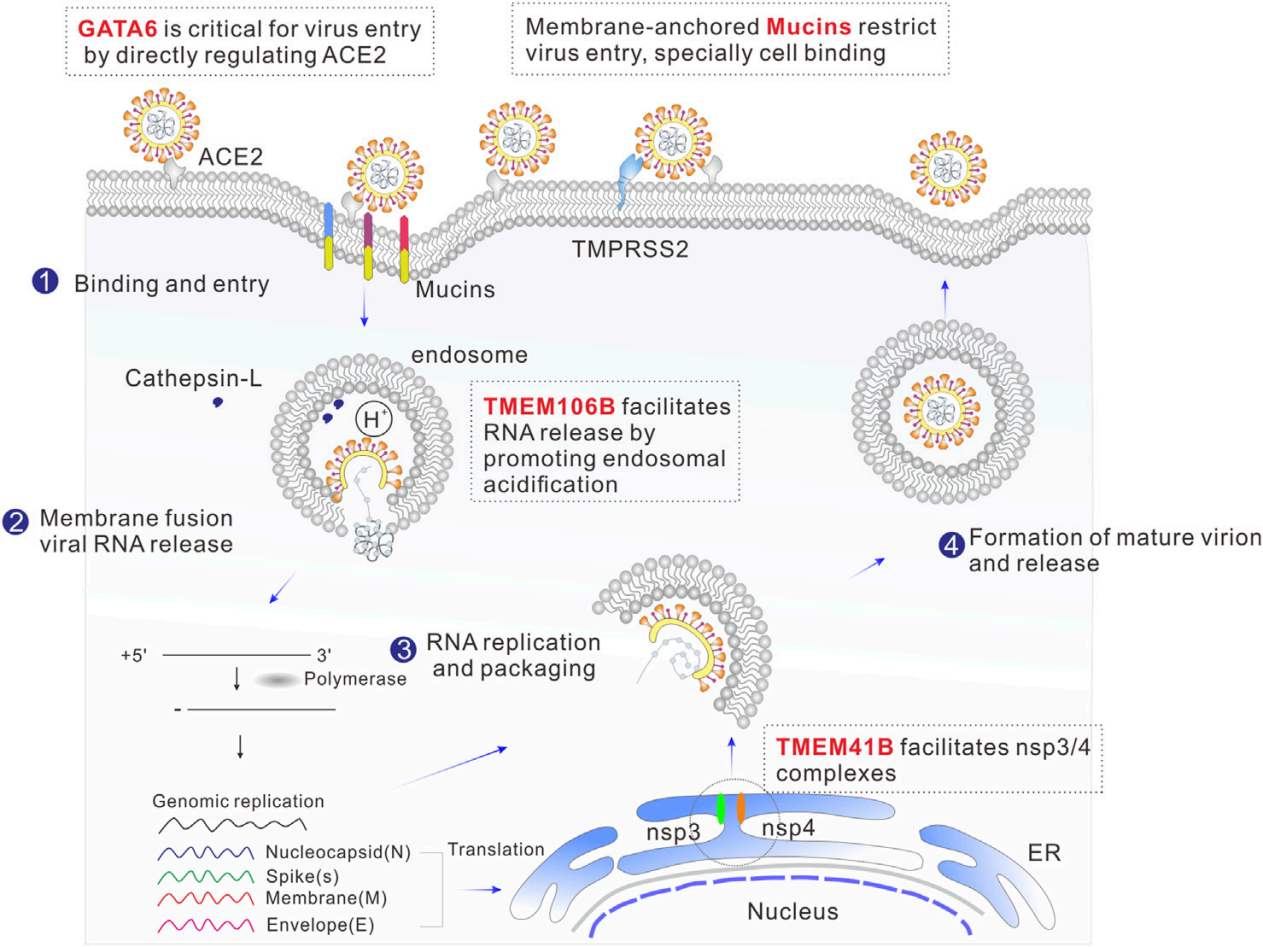
Schematic illustration of the SARS-CoV-2 life cycle, including binding, membrane fusion, uncoating, RNA replication, transcription, translation, packaging, and release. The top-ranked host genes from the CRISPR screen that are involved in viral replication cycle are highlighted in red.

RNA interference (RNAi) and Clustered Regularly Interspaced Short Palindromic Repeats (CRISPR) have proven effective in identifying host factors essential for infection by various viruses (Puschnik et al., 2017). CRISPR/Cas9-based screening platforms, such as loss-of-function CRISPR knockout (CRISPRKO) and gain-of-function CRISPR activation (CRISPRa), have emerged as the crucial methods for pinpointing host genes crucial for SARS-CoV-2 infection (Baggen et al., 2021; Daniloski et al., 2021; Hoffmann et al., 2021; Schneider et al., 2021; Wang et al., 2021; Wei et al., 2021; Biering et al., 2022; Israeli et al., 2022; Rebendenne et al., 2022). These cutting-edge techniques are driving the development of novel therapeutic strategies aimed at eradiating the disease. During each step of the viral life cycle, specific cellular proteins are hijacked and play crucial roles; for example, previous studies have shown that angiotensin-converting enzyme 2 (ACE2) is exploited as the viral entry receptor, additionally, cellular proteases, such as Transmembrane Serine Protease 2 (TMPRSS2), Cathepsin L (Ctsl), and furin, are important for the activation of the viral spike(S) protein (Benton et al., 2020; Hoffmann et al., 2020). Significantly, recent CRISPR-based screens have identified several novel host factors involved in coronavirus infection. The fact that well-known viral entry receptors or cofactors, such as ACE2, TMPRSS2 and cathepsin L, are ranked among the top list of CRISPR screenings (Wei et al., 2021; Biering et al., 2022; Rebendenne et al., 2022), underscores the value of this methodology in discovering the host factors involved. Herein, we summarize recent findings on some of the top-ranked host factors, especially roles in virus-host interaction and implications for antiviral therapeutics (Figure 1).

## TMEM41B

Genome-scale CRISPR-Cas9 knockout screens were performed using human Huh-7.5 (.1) (Schneider et al., 2021; Wang et al., 2021) or Huh7 (Baggen et al., 2021) hepatoma cells to identify Transmembrane protein 41B (TMEM41B) as a host factor for SARS-CoV-2. Additionally, utilizing same screening method, TMEM41B remain among the top hits in Huh7 cell lines for Middle East Respiratory Syndrome Coronavirus (MERS-CoV) and seasonal alpha-coronavirus Human coronaviruses (HCoV-229E) (Kratzel et al., 2021; Trimarco et al., 2021). It has also been identified in B3GALT6-deficient human haploid (HAP1) cells for Zika and Yellow Fever Viruses (Hoffmann et al., 2021). These findings reinforce the notion that TMEM41B serves as a broad-spectrum host factor for infection caused by coronaviruses and other types of viruses (Baggen et al., 2021; Hoffmann et al., 2021; Kratzel et al., 2021; Schneider et al., 2021; Trimarco et al., 2021; Wang et al., 2021). TMEM41B is integral endoplasmic reticulum (ER)-located transmembrane protein whose physiological function is currently not well understood. Previous research has demonstrated that this protein is not only crucial for synaptic transmission in motor circuit neurons (Imlach et al., 2012; Lotti et al., 2012), but also essential for the autophagy pathway, as it is involved in the early stages of autophagosome formation and plays a key role in intracellular lipid mobilization (Moretti et al., 2018; Morita et al., 2019; Shoemaker et al., 2019). Mechanistic studies of the role of TMEM41B in SARS-CoV-2 infection have revealed that, like the flavivirus replication cycle (Hoffmann et al., 2021), TMEM41B is involved in post-entry to facilitate the ER membrane remodeling essential for forming replication organelles (Schneider et al., 2021). The most prominent replication organelles induced by SARS-CoV-2 infection are double-membrane vesicles (DMVs), which are formed by reshaping the endomembrane in the host cells (Baggen et al., 2021; Hoffmann et al., 2021; Sun et al., 2021; Trimarco et al., 2021; Ji et al., 2022). Facilitating the formation of nsp3/4 complexes is thought to be the critical mechanism by which TMEM41B contributes to the DMV biogenesis, as these complexes are able to create curvature in the ER through “zipping” interactions (Ji et al., 2022). Another study has shown that TMEM41B may contribute to the formation of viral replication complexes by mobilizing cholesterol and other lipids, which in turn facilitates the expansion and curvature of host membranes (Trimarco et al., 2021). Importantly, the function of TMEM41B is validated *in vitro*; for example, the infectivity of SARS-CoV-2 was strongly decreased in Huh 7.5 and A549^ACE2/TMPRSS2^ cells by genetic deletion of TMEM41B. Conversely, the reintroduction of TMEM41B cDNA restored the infectivity of SARS-CoV-2 in these cell lines (Schneider et al., 2021).

## TMEM106B

The lysosomal transmembrane protein TMEM106B was identified as notable host factor in multiple Genome-wide CRISPR based genetic screens in Huh7 (Baggen et al., 2021) and Huh7.5.1 (Schneider et al., 2021; Wang et al., 2021) cells required for SARS-CoV-2 infection. TMEM106B is a 274 amino acid transmembrane protein that is located in late endosomes and lysosomes. It is not well characterized and has only recently gained attention due to its involvement in frontotemporal dementia, which is the second leading cause of pre-senile neurodegeneration. TMEM106B plays a crucial role in regulating various aspects of lysosome function, including size, number, mobility, and trafficking (Luningschror et al., 2020). Its importance lies in its pivotal role in lysosome acidification, achieved through its interaction with the proton pump vacuolar, ATPase accessory protein 1 (Klein et al., 2017). Previous reports have indicated that SARS-CoV-2 S-pseudotyped virus entry requires endosomal acidification (Hoffmann et al., 2020; Ou et al., 2020). Furthermore, overexpression of TMEM106B specifically enhances cell entry by pseudoviruses carrying SARS-CoV-2 spike protein, and it has been observed that TMEM106B has a high-level expression in airway epithelium from patients with COVID-19 compared to non-infected patients (Baggen et al., 2021). Based on these findings, it is believed that the expression of TMEM106B increases susceptibility to SARS-CoV-2 by promoting endosomal acidification or acting as an endosomal cofactor, thereby facilitating the delivery of the SARS-CoV-2 genome into the cytoplasm (Baggen et al., 2021). This was further supported by the fact that genetic depletion of TMEM106B decreased SARS-CoV-2 infection in Huh7.5.1, Huh7, Hep3B, NCI-H2110, A549, NCI-H1975, as well as primary bronchial epithelial cells (HBECs), and this effect was reversed by complementing with TMEM106B cDNA indicating the specificity and efficiency of this gene target (Baggen et al., 2021; Wang et al., 2021). Interestingly, the high expression of TMEM106B expression in the brain compared to the lung might contribute to neurological symptoms such as stroke, brain hemorrhage and memory loss in COVID-19 patients (Uhlen et al., 2015; Varatharaj et al., 2020). Therefore, TMEM106B targeting may prevent neurological symptoms associated with COVID-19.

## GATA6

CRISPR-based genome-wide gene knockout screen was performed to identified GATA binding protein 6 (GATA6) scored as the second strongest proviral factor in the human lung epithelial cell line Calu-3 in response to SARS-CoV-2 (Israeli et al., 2022). GATA6 is a member of a small family of zinc finger DNA-binding transcription factor that play an important role in the regulation of visceral endoderm differentiation and it is the only GATA family member expressed in the distal epithelium of the developing lung (Yang et al., 2002). GATA6 was upregulated in SARS-CoV-2 infected lung and has been reported to be critical for SARS-CoV-2 cell entry by binding to the ACE2 promoter and directly regulating its transcription (Islam and Khan, 2020; Israeli et al., 2022). The manipulation of GATA6 targeting through CRISPR abrogation provides protection to Calu-3 cells from SARS-CoV-2 infection as well as other variants of concern (VOCs), including Alpha, Beta and Delta (Israeli et al., 2022). Elevated expression of GATA6 has been observed in COVID-19 patients compared with healthy individuals indicating the clinical relevance of GATA6 to SARS-CoV-2 infection.

## Mucins

Membrane associated mucins were identified in lung epithelial Calu-3 cell line for SARS-CoV-2 infection through gain-of-function CRISPRa screen (Biering et al., 2022; Rebendenne et al., 2022). Mucins are a family of high molecular weight *O*-glycosylated glycoproteins and are the primary constituent of mucus lining the epithelial tract of the lungs and gut (Lillehoj et al., 2013). Mucins can be divided into two types: transmembrane mucins, such as MUC1 or MUC4, and secreted, gel-forming mucins, such as MUC5AC and MUC5B. They have a well-established role in host defense against pathogens (McAuley et al., 2017; Chatterjee et al., 2020). Previous studies have reported that at cell level, CRISPR-mediated overexpression mucins (MCU1, MCU4, MUC21) had a potent impact on decreasing SARS-CoV-2 replication in Calu3 cells (Rebendenne et al., 2022). Moreover, when endogenous mucins are digested with protease, the cells become more permissive to SARS-CoV-2 infection (Biering et al., 2022). Additionally, mucins have been shown to restrict infection of multiple SARS-CoV-2 variants, including alpha (B.1.1.7), beta (B.1.351), gamma (P.1), epsilon (B.1.429) and WA/1(Biering et al., 2022). Furthermore, at tissue level, it has been observed that all four transmembrane mucins (MCU1, MCU4, MUC13, MUC21) are upregulated in SARS -CoV-2 infected human lung tissue, and MCU1, MCU4 are increased in infected hamster and mouse lung tissue (Biering et al., 2022). All these suggested that mucins may serve as antiviral host receptors. Moreover, significant upregulation consistent with a protective role was detected in the epithelial cell fraction of human bronchoalveolar lavage fluid (BALF) from patients with SARS-CoV-2 infection (Biering et al., 2022). The evidence that mucins play a protective role against SARS-CoV-2 infection *in vivo* is supported by the fact that a triple membrane-anchored mucin KO mouse (Muc1^−/−^/Muc4^−/−^/Muc16^−/−^) exhibited a higher level of SARS-CoV-2 N protein and RNA, as well as a higher viral titer, when compared to wild-type control mice (Biering et al., 2022). Importantly, it was believed that mucins affect the step of cell binding to restrict SARS-CoV-2 entry (Biering et al., 2022). In contrast to transmembrane mucins, gel-forming mucins such as MUC5AC have been shown to play a proviral role in SARS-CoV-2 infection (Biering et al., 2022).

## CRISPR/Cas13-based gene therapy to target host factors

**FIGURE 2:**
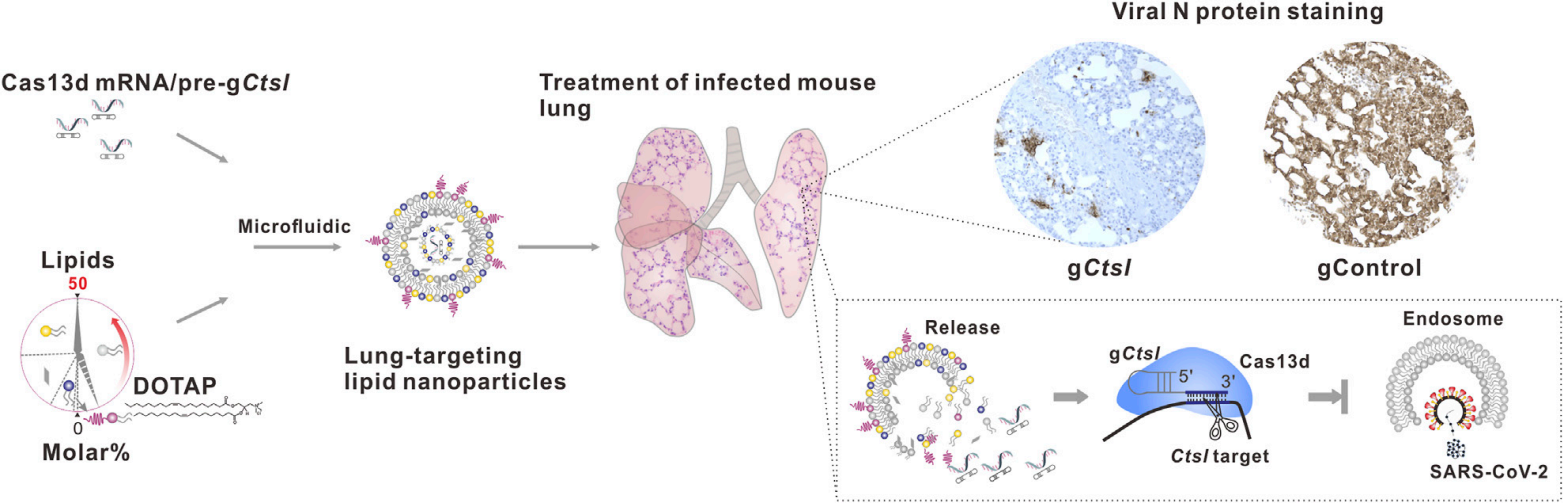
Schematic depiction of lung-targeting LNPs encapsulating CRISPR/Cas13d mRNA/pre-g*Ctsl* demonstrated efficacy in controlling of SARS-CoV-2 infection.

Targeting host proteases represents a viable strategy for preventing and treating COVID-19. However, despite several host protease inhibitors showing efficacy in blocking the entry of coronaviruses (e.g., SARS-CoV-2 and SARS-CoV-1) *in vitro*, their therapeutic effects in animal model have been limited (Zhou et al., 2015; Liu et al., 2020). Gene therapy using CRISPR/Cas9, a powerful tool for targeted gene editing, provides a potential alternative method for targeting host proteases. However, the possibility of introducing unwanted irreversible DNA changes using CRISPR/Cas9 is a major obstacle to its therapeutic application (Pummed, 2018). The CRISPR/Cas13d (CasRx) RNA targeting system offers a way to transiently knockdown host proteases at the mRNA level without causing off-target effects that are typical of RNA interference (RNAi) strategies (Birmingham et al., 2006; Sigoillot et al., 2012; Konermann et al., 2018). CasRx is highly efficient and specific in RNA knockdown, and RNA interference effects have not been reported for Cas13 guide RNAs in mammalian cells (Cox et al., 2017; Konermann et al., 2018). The small size of the CasRx enzyme makes it suitable for packaging into an adeno-associated (AAV) vector (Konermann et al., 2018). However, clinical application of AAV vectors is limited because of the viral immunogenicity, the small percentage of cells targeting, viral production difficulties, and tumorigenic concern (Thomas et al., 2003; Hardee et al., 2017; Colella et al., 2018; Nguyen et al., 2020). Lipid nanoparticles (LNPs) have been successfully employed in clinical settings for the delivery of therapeutic agents and vaccines (Mullard, 2018; Jackson et al., 2020). We have recently developed chemically engineered LNPs that encapsulate CRISPR/Cas13d, enabling effective control of SARS-CoV-2 infection by specifically targeting a robust host factor known as Ctsl (Figure 2) (Cui et al., 2022). Ctsl is an important endosomal cysteine protease that faciliates viral entry through priming the virus endosome membrane fusion (Liu et al., 2020). This approach demonstrated the ability to extend the survival of mice that were lethally infected with SARS-CoV-2, by reducing the viral load in the lungs, suppressing the expression of proinflammatory cytokines/chemokines, and mitigating the severity of pulmonary interstitial inflammation. Importantly, the effectiveness of post-infection treatment suggests the CRISPR could be a potential treatment for SARS-CoV-2 (Cui et al., 2022).

## Conclusion and future perspectives

In the past two decades, three human beta-coronaviruses have emerged and caused outbreaks that have generated significant global health concerns. Among these viruses, SARS-CoV and Middle East respiratory syndrome (MERS) have relatively high fatality rates, with MERS still sporadic, while SARS-CoV-2 is the most recent and widespread coronavirus to cause a global pandemic. This virus has resulted in millions of deaths and substantial morbidity worldwide, making it a major public health crisis. Since the beginning of the COVID-19 pandemic at the end of 2019, the identification of host factors has become a central focus of the biomedical research community. Genome-wide CRISPR/Cas9-based screening has been instrumental in identifying novel host factors of viral pathogens (Daniloski et al., 2021; Wei et al., 2021). By comprehending the host factors necessary for viral replication and infection, we can identify potential targets for developing new antiviral therapies or repurposing existing drugs (Puschnik et al., 2017; Cui et al., 2022). In this review we have summarized several newly identified host factors, including TMEM41B, TMEM106B, GATA6, and mucins. The identification and characterization of these host factors will provide valuable insights into host-targeted therapeutic strategies for SARS-CoV-2 and related coronavirus outbreaks in the future.

Amid the ongoing fight against the SARS-CoV-2 infection, a range of vaccines—including Pfizer and Moderna’s mRNA vaccines—and medications such as remdesivir and paxlovid, have been granted emergency use authorization to save lives. Developing vaccines and drugs is typically a time-consuming endeavor, and the rapid emergence of SARS-CoV-2 variants may undermine the effectiveness of existing treatments. Given that infections caused by SARS-CoV-2, including its variants, as well as other coronaviruses, critically depend on host factors, some of which are not amenable to small molecule strategies, utilizing the CRISPR/Cas system—particularly the reversible and specific RNA-targeting CRISPR/Cas13—offers substantial promise in the fight against current and future coronavirus infections. Compared to DNA targeting/editing, the reversible and flexible characteristics of Cas13-based RNA-targeting/editing have the potential to alleviate existing ethical barriers, such as those concerning the impact on future descendants and the therapeutic genome editing of late-onset disorders. This, in turn, opens up new avenues for safe clinical interventions, thereby expanding the realm of possibilities (Gold et al., 2021). However, the specificity of Cas13 effectors in mammalian cells remains a topic of debate. This originates from the scenario where, upon binding to a target RNA, the Cas13 complex undergoes a conformational change. This change activates the nuclease domains that can be exposed on the surface of the complex, leading not only to the cleavage of the target RNA, but also to the incidental cleavage of bystander RNAs—a phenomenon referred to as collateral activity, which is commonly observed in bacterial systems (Abudayyeh et al., 2016; East-Seletsky et al., 2016). Our group, alongside other groups, has consistently observed no evidence of this activity in eukaryotic cells across various experiments (Abudayyeh et al., 2017; Konermann et al., 2018; Huynh et al., 2020; Kushawah et al., 2020; Cui et al., 2022), and the extensive utilization of Cas13 in other studies further supports its safety and absence of such activity (Cox et al., 2017; He et al., 2020; Wessels et al., 2020; Li et al., 2021). Conversely, preliminary yet limited evidence has started to suggest that collateral activity might occur in certain mammalian cells when targeting specific RNAs (Ozcan et al., 2021; Xu et al., 2021; Kelley et al., 2022; Li et al., 2023; Shi et al., 2023). This introduces an element of doubt regarding the practical implementation of Cas13-based therapeutics. Interestingly, the recently developed CRISPR-Csm complexes, a multi-protein effector from type III CRISPR systems, presents itself as an appealing RNA target tool in eukaryotic cells with minimal off-target effects, providing renewed hope for the effective and safe utilization of CRISPR RNA-targeting in the future (Colognori et al., 2023). Finally, it is crucial to mention the need for the advancement of LNP delivery systems, designed specifically to selectively deliver the CRISPR RNA-targeting tool to the upper and lower respiratory systems. Although the current Selective Organ Targeting (SORT) LNP delivery system has shown promising results in a limited range of tissues, including the lungs (Cheng et al., 2020), the practical application of this technology calls for further enhancements to ensure the effective and safe delivery. In summary, the LNP-CRISPR RNA-targeting approach must undergo rigorous efficacy and safety assessments before it can be responsibly considered for human application.

## References

[B1] Abudayyeh O. O. Gootenberg J. S. Essletzbichler P. Han S. Joung J. Belanto J. J. (2017). RNA targeting with CRISPR-Cas13. Nature 550, 280–284. 10.1038/nature24049 28976959 PMC5706658

[B2] Abudayyeh O. O. Gootenberg J. S. Konermann S. Joung J. Slaymaker I. M. Cox D. B. T. (2016). C2c2 is a single-component programmable RNA-guided RNA-targeting CRISPR effector. Science 353, aaf5573. 10.1126/science.aaf5573 27256883 PMC5127784

[B3] Baggen J. Persoons L. Vanstreels E. Jansen S. Van Looveren D. Boeckx B. (2021). Genome-wide CRISPR screening identifies TMEM106B as a proviral host factor for SARS-CoV-2. Nat. Genet. 53, 435–444. 10.1038/s41588-021-00805-2 33686287

[B4] Benton D. J. Wrobel A. G. Xu P. Roustan C. Martin S. R. Rosenthal P. B. (2020). Receptor binding and priming of the spike protein of SARS-CoV-2 for membrane fusion. Nature 588, 327–330. 10.1038/s41586-020-2772-0 32942285 PMC7116727

[B5] Biering S. B. Sarnik S. A. Wang E. Zengel J. R. Leist S. R. Schäfer A. (2022). Genome-wide bidirectional CRISPR screens identify mucins as host factors modulating SARS-CoV-2 infection. Nat. Genet. 54, 1078–1089. 10.1038/s41588-022-01131-x 35879412 PMC9355872

[B6] Birmingham A. Anderson E. M. Reynolds A. Ilsley-Tyree D. Leake D. Fedorov Y. (2006). 3' UTR seed matches, but not overall identity, are associated with RNAi off-targets. Nat. Methods 3, 199–204. 10.1038/nmeth854 16489337

[B7] Chatterjee M. van Putten J. P. M. Strijbis K. (2020). Defensive properties of mucin glycoproteins during respiratory infections-relevance for SARS-CoV-2. mBio 11, e02374-20. 10.1128/mBio.02374-20 33184103 PMC7663010

[B8] Cheng Q. Wei T. Farbiak L. Johnson L. T. Dilliard S. A. Siegwart D. J. (2020). Selective organ targeting (SORT) nanoparticles for tissue-specific mRNA delivery and CRISPR-Cas gene editing. Nat. Nanotechnol. 15, 313–320. 10.1038/s41565-020-0669-6 32251383 PMC7735425

[B9] Colella P. Ronzitti G. Mingozzi F. (2018). Emerging issues in AAV-mediated *in vivo* gene therapy. Mol. Ther. Methods Clin. Dev. 8, 87–104. 10.1016/j.omtm.2017.11.007 29326962 PMC5758940

[B10] Colognori D. Trinidad M. Doudna J. A. (2023). Precise transcript targeting by CRISPR-Csm complexes. Nat. Biotechnol. 10.1038/s41587-022-01649-9 PMC10497410 36690762

[B11] Cox D. B. T. Gootenberg J. S. Abudayyeh O. O. Franklin B. Kellner M. J. Joung J. (2017). RNA editing with CRISPR-Cas13. Science 358, 1019–1027. 10.1126/science.aaq0180 29070703 PMC5793859

[B12] Cui Z. Zeng C. Huang F. Yuan F. Yan J. Zhao Y. (2022). Cas13d knockdown of lung protease Ctsl prevents and treats SARS-CoV-2 infection. Nat. Chem. Biol. 18, 1056–1064. 10.1038/s41589-022-01094-4 35879545 PMC10082993

[B13] Daniloski Z. Jordan T. X. Wessels H. H. Hoagland D. A. Kasela S. Legut M. (2021). Identification of required host factors for SARS-CoV-2 infection in human cells. Cell 184, 92–105.e16. 10.1016/j.cell.2020.10.030 33147445 PMC7584921

[B14] East-Seletsky A. O'Connell M. R. Knight S. C. Burstein D. Cate J. H. D. Tjian R. (2016). Two distinct RNase activities of CRISPR-C2c2 enable guide-RNA processing and RNA detection. Nature 538, 270–273. 10.1038/nature19802 27669025 PMC5576363

[B15] Fehr A. R. Perlman S. (2015). Coronaviruses: An overview of their replication and pathogenesis. Methods Mol. Biol. 1282, 1–23. 10.1007/978-1-4939-2438-7_1 25720466 PMC4369385

[B16] Fung T. S. Liu D. X. (2019). Human coronavirus: Host-pathogen interaction. Annu. Rev. Microbiol. 73, 529–557. 10.1146/annurev-micro-020518-115759 31226023

[B17] Gold A. Levanon E. Y. Eisenberg E. (2021). The new RNA-editing era - ethical considerations. Trends Genet. 37, 685–687. 10.1016/j.tig.2021.04.013 33975753

[B18] Hardee C. L. Arevalo-Soliz L. M. Hornstein B. D. Zechiedrich L. (2017). Advances in non-viral DNA vectors for gene therapy. Genes (Basel) 8, 65. 10.3390/genes8020065 28208635 PMC5333054

[B19] He B. Peng W. Huang J. Zhang H. Zhou Y. Yang X. (2020). Modulation of metabolic functions through Cas13d-mediated gene knockdown in liver. Protein Cell 11, 518–524. 10.1007/s13238-020-00700-2 32185621 PMC7095259

[B20] Hoffmann H. H. Schneider W. M. Rozen-Gagnon K. Miles L. A. Schuster F. Razooky B. (2021). TMEM41B is a pan-flavivirus host factor. Cell 184, 133–148.e20. 10.1016/j.cell.2020.12.005 33338421 PMC7954666

[B21] Hoffmann M. Kleine-Weber H. Schroeder S. Krüger N. Herrler T. Erichsen S. (2020). SARS-CoV-2 cell entry depends on ACE2 and TMPRSS2 and is blocked by a clinically proven protease inhibitor. Cell 181, 271–280.e8. 10.1016/j.cell.2020.02.052 32142651 PMC7102627

[B22] Huynh N. Depner N. Larson R. King-Jones K. (2020). A versatile toolkit for CRISPR-Cas13-based RNA manipulation in Drosophila. Genome Biol. 21, 279. 10.1186/s13059-020-02193-y 33203452 PMC7670108

[B23] Imlach W. L. Beck E. S. Choi B. J. Lotti F. Pellizzoni L. McCabe B. D. (2012). SMN is required for sensory-motor circuit function in Drosophila. Cell 151, 427–439. 10.1016/j.cell.2012.09.011 23063130 PMC3475188

[B24] Islam A. Khan M. A. (2020). Lung transcriptome of a COVID-19 patient and systems biology predictions suggest impaired surfactant production which may be druggable by surfactant therapy. Sci. Rep. 10, 19395. 10.1038/s41598-020-76404-8 33173052 PMC7656460

[B25] Israeli M. Finkel Y. Yahalom-Ronen Y. Paran N. Chitlaru T. Israeli O. (2022). Genome-wide CRISPR screens identify GATA6 as a proviral host factor for SARS-CoV-2 via modulation of ACE2. Nat. Commun. 13, 2237. 10.1038/s41467-022-29896-z 35469023 PMC9039069

[B26] Jackson L. A. Anderson E. J. Rouphael N. G. Roberts P. C. Makhene M. Coler R. N. (2020). An mRNA vaccine against SARS-CoV-2 - preliminary report. N. Engl. J. Med. 383, 1920–1931. 10.1056/NEJMoa2022483 32663912 PMC7377258

[B27] Ji M. Li M. Sun L. Zhao H. Li Y. Zhou L. (2022). VMP1 and TMEM41B are essential for DMV formation during beta-coronavirus infection. J. Cell Biol. 221, e202112081. 10.1083/jcb.202112081 35536318 PMC9097365

[B28] Kelley C. P. Haerle M. C. Wang E. T. (2022). Negative autoregulation mitigates collateral RNase activity of repeat-targeting CRISPR-Cas13d in mammalian cells. Cell Rep. 40, 111226. 10.1016/j.celrep.2022.111226 35977479 PMC9809062

[B29] Klein Z. A. Takahashi H. Ma M. Stagi M. Zhou M. Lam T. T. (2017). Loss of TMEM106B ameliorates lysosomal and frontotemporal dementia-related phenotypes in progranulin-deficient mice. Neuron 95, 281–296.e6. 10.1016/j.neuron.2017.06.026 28728022 PMC5558861

[B30] Konermann S. Lotfy P. Brideau N. J. Oki J. Shokhirev M. N. Hsu P. D. (2018). Transcriptome engineering with RNA-targeting type VI-D CRISPR effectors. Cell 173, 665–676.e14. 10.1016/j.cell.2018.02.033 29551272 PMC5910255

[B31] Kratzel A. Kelly J. N. V'kovski P. Portmann J. Brüggemann Y. Todt D. (2021). A genome-wide CRISPR screen identifies interactors of the autophagy pathway as conserved coronavirus targets. PLoS Biol. 19, e3001490. 10.1371/journal.pbio.3001490 34962926 PMC8741300

[B32] Kushawah G. Hernandez-Huertas L. Abugattas-Nuñez Del Prado J. Martinez-Morales J. R. DeVore M. L. Hassan H. (2020). CRISPR-Cas13d induces efficient mRNA knockdown in animal embryos. Dev. Cell 54, 805–817.e7. 10.1016/j.devcel.2020.07.013 32768421

[B33] Li S. Li X. Xue W. Zhang L. Yang L. Z. Cao S. M. (2021). Screening for functional circular RNAs using the CRISPR-Cas13 system. Nat. Methods 18, 51–59. 10.1038/s41592-020-01011-4 33288960

[B34] Liu T. Luo S. Libby P. Shi G. P. (2020). Cathepsin L-selective inhibitors: A potentially promising treatment for COVID-19 patients. Pharmacol. Ther. 213, 107587. 10.1016/j.pharmthera.2020.107587 32470470 PMC7255230

[B35] Li Y. Xu J. Guo X. Li Z. Cao L. Liu S. (2023). The collateral activity of RfxCas13d can induce lethality in a RfxCas13d knock-in mouse model. Genome Biol. 24, 20. 10.1186/s13059-023-02860-w 36726140 PMC9893547

[B36] Lillehoj E. P. Kato K. Lu W. Kim K. C. (2013). Cellular and molecular biology of airway mucins. Int. Rev. Cell Mol. Biol. 303, 139–202. 10.1016/B978-0-12-407697-6.00004-0 23445810 PMC5593132

[B37] Lotti F. Imlach W. L. Saieva L. Beck E. S. Hao L. T. Li D. K. (2012). An SMN-dependent U12 splicing event essential for motor circuit function. Cell 151, 440–454. 10.1016/j.cell.2012.09.012 23063131 PMC3474596

[B38] Luningschror P. Werner G. Stroobants S. Kakuta S. Dombert B. Sinske D. (2020). The FTLD risk factor TMEM106B regulates the transport of lysosomes at the axon initial segment of motoneurons. Cell Rep. 30, 3506–3519.e6. 10.1016/j.celrep.2020.02.060 32160553

[B39] McAuley J. L. Corcilius L. Tan H. X. Payne R. J. McGuckin M. A. Brown L. E. (2017). The cell surface mucin MUC1 limits the severity of influenza A virus infection. Mucosal Immunol. 10, 1581–1593. 10.1038/mi.2017.16 28327617

[B40] Moretti F. Bergman P. Dodgson S. Marcellin D. Claerr I. Goodwin J. M. (2018). TMEM41B is a novel regulator of autophagy and lipid mobilization. EMBO Rep. 19, e45889. 10.15252/embr.201845889 30126924 PMC6123663

[B41] Morita K. Hama Y. Mizushima N. (2019). TMEM41B functions with VMP1 in autophagosome formation. Autophagy 15, 922–923. 10.1080/15548627.2019.1582952 30773971 PMC6526808

[B42] Mullard A. (2018). FDA approves landmark RNAi drug. Nat. Rev. Drug Discov. 17, 613. 10.1038/nrd.2018.152 30160250

[B43] Nguyen G. N. Everett J. K. Kafle S. Roche A. M. Raymond H. E. Leiby J. (2020). A long-term study of AAV gene therapy in dogs with hemophilia A identifies clonal expansions of transduced liver cells. Nat. Biotechnol. 39, 47–55. 10.1038/s41587-020-0741-7 33199875 PMC7855056

[B44] Ou X. Liu Y. Lei X. Li P. Mi D. Ren L. (2020). Characterization of spike glycoprotein of SARS-CoV-2 on virus entry and its immune cross-reactivity with SARS-CoV. Nat. Commun. 11, 1620. 10.1038/s41467-020-15562-9 32221306 PMC7100515

[B45] Ozcan A. Krajeski R. Ioannidi E. Lee B. Gardner A. Makarova K. S. (2021). Programmable RNA targeting with the single-protein CRISPR effector Cas7-11. Nature 597, 720–725. 10.1038/s41586-021-03886-5 34489594

[B46] Pummed (2018). Keep off-target effects in focus. Nat. Med. 24, 1081. 10.1038/s41591-018-0150-3 30082857

[B47] Puschnik A. S. Majzoub K. Ooi Y. S. Carette J. E. (2017). A CRISPR toolbox to study virus-host interactions. Nat. Rev. Microbiol. 15, 351–364. 10.1038/nrmicro.2017.29 28420884 PMC5800792

[B48] Rebendenne A. Roy P. Bonaventure B. Chaves Valadão A. L. Desmarets L. Arnaud-Arnould M. (2022). Bidirectional genome-wide CRISPR screens reveal host factors regulating SARS-CoV-2, MERS-CoV and seasonal HCoVs. Nat. Genet. 54, 1090–1102. 10.1038/s41588-022-01110-2 35879413 PMC11627114

[B49] Schneider W. M. Luna J. M. Hoffmann H. H. Sánchez-Rivera F. J. Leal A. A. Ashbrook A. W. (2021). Genome-scale identification of SARS-CoV-2 and pan-coronavirus host factor networks. Cell 184, 120–132.e14. 10.1016/j.cell.2020.12.006 33382968 PMC7796900

[B50] Shi P. Murphy M. R. Aparicio A. O. Kesner J. S. Fang Z. Chen Z. (2023). Collateral activity of the CRISPR/RfxCas13d system in human cells. Commun. Biol. 6, 334. 10.1038/s42003-023-04708-2 36977923 PMC10049998

[B51] Shoemaker C. J. Huang T. Q. Weir N. R. Polyakov N. J. Schultz S. W. Denic V. (2019). CRISPR screening using an expanded toolkit of autophagy reporters identifies TMEM41B as a novel autophagy factor. PLoS Biol. 17, e2007044. 10.1371/journal.pbio.2007044 30933966 PMC6459555

[B52] Sigoillot F. D. Lyman S. Huckins J. F. Adamson B. Chung E. Quattrochi B. (2012). A bioinformatics method identifies prominent off-targeted transcripts in RNAi screens. Nat. Methods 9, 363–366. 10.1038/nmeth.1898 22343343 PMC3482495

[B53] Sun L. Zhao C. Fu Z. Fu Y. Su Z. Li Y. (2021). Genome-scale CRISPR screen identifies TMEM41B as a multi-function host factor required for coronavirus replication. PLoS Pathog. 17, e1010113. 10.1371/journal.ppat.1010113 34871328 PMC8675922

[B54] Thomas C. E. Ehrhardt A. Kay M. A. (2003). Progress and problems with the use of viral vectors for gene therapy. Nat. Rev. Genet. 4, 346–358. 10.1038/nrg1066 12728277

[B55] Trimarco J. D. Heaton B. E. Chaparian R. R. Burke K. N. Binder R. A. Gray G. C. (2021). TMEM41B is a host factor required for the replication of diverse coronaviruses including SARS-CoV-2. PLoS Pathog. 17, e1009599. 10.1371/journal.ppat.1009599 34043740 PMC8189496

[B56] Uhlen M. Fagerberg L. Hallström B. M. Lindskog C. Oksvold P. Mardinoglu A. (2015). Proteomics. Tissue-based map of the human proteome. Science 347, 1260419. 10.1126/science.1260419 25613900

[B57] Varatharaj A. Thomas N. Ellul M. A. Davies N. W. S. Pollak T. A. Tenorio E. L. (2020). Neurological and neuropsychiatric complications of COVID-19 in 153 patients: A UK-wide surveillance study. Lancet Psychiatry 7, 875–882. 10.1016/S2215-0366(20)30287-X 32593341 PMC7316461

[B58] Wang M. Y. Zhao R. Gao L. J. Gao X. F. Wang D. P. Cao J. M. (2020). SARS-CoV-2: Structure, biology, and structure-based therapeutics development. Front. Cell Infect. Microbiol. 10, 587269. 10.3389/fcimb.2020.587269 33324574 PMC7723891

[B59] Wang R. Simoneau C. R. Kulsuptrakul J. Bouhaddou M. Travisano K. A. Hayashi J. M. (2021). Genetic screens identify host factors for SARS-CoV-2 and common cold coronaviruses. Cell 184, 106–119.e14. 10.1016/j.cell.2020.12.004 33333024 PMC7723770

[B60] Wei J. Alfajaro M. M. DeWeirdt P. C. Hanna R. E. Lu-Culligan W. J. Cai W. L. (2021). Genome-wide CRISPR screens reveal host factors critical for SARS-CoV-2 infection. Cell 184, 76–91 e13. 10.1016/j.cell.2020.10.028 33147444 PMC7574718

[B61] Wessels H. H. Méndez-Mancilla A. Guo X. Legut M. Daniloski Z. Sanjana N. E. (2020). Massively parallel Cas13 screens reveal principles for guide RNA design. Nat. Biotechnol. 38, 722–727. 10.1038/s41587-020-0456-9 32518401 PMC7294996

[B62] Xu C. Zhou Y. Xiao Q. He B. Geng G. Wang Z. (2021). Programmable RNA editing with compact CRISPR-Cas13 systems from uncultivated microbes. Nat. Methods 18, 499–506. 10.1038/s41592-021-01124-4 33941935

[B63] Yang H. Lu M. M. Zhang L. Whitsett J. A. Morrisey E. E. (2002). GATA6 regulates differentiation of distal lung epithelium. Development 129, 2233–2246. 10.1242/dev.129.9.2233 11959831

[B64] Zhou Y. Vedantham P. Lu K. Agudelo J. Carrion R. Jr Nunneley J. W. (2015). Protease inhibitors targeting coronavirus and filovirus entry. Antivir. Res. 116, 76–84. 10.1016/j.antiviral.2015.01.011 25666761 PMC4774534

